# Imaging the dynamics of transcription loops in living chromosomes

**DOI:** 10.1007/s00412-018-0667-8

**Published:** 2018-04-03

**Authors:** Garry T. Morgan

**Affiliations:** School of Life Sciences, University of Nottingham, Queens Medical Centre, Nottingham, NG7 2UH UK

**Keywords:** Lampbrush chromosomes, CELF1, Nascent RNP, Transcription unit, Nuclear compartment

## Abstract

When in the lampbrush configuration, chromosomes display thousands of visible DNA loops that are transcribed at exceptionally high rates by RNA polymerase II (pol II). These transcription loops provide unique opportunities to investigate not only the detailed architecture of pol II transcription sites but also the structural dynamics of chromosome looping, which is receiving fresh attention as the organizational principle underpinning the higher-order structure of all chromosome states. The approach described here allows for extended imaging of individual transcription loops and transcription units under conditions in which loop RNA synthesis continues. In intact nuclei from lampbrush-stage *Xenopus* oocytes isolated under mineral oil, highly specific targeting of fluorescent fusions of the RNA-binding protein CELF1 to nascent transcripts allowed functional transcription loops to be observed and their longevity assessed over time. Some individual loops remained extended and essentially static structures over time courses of up to an hour. However, others were less stable and shrank markedly over periods of 30–60 min in a manner that suggested that loop extension requires continued dense coverage with nascent transcripts. In stable loops and loop-derived structures, the molecular dynamics of the visible nascent RNP component were addressed using photokinetic approaches. The results suggested that CELF1 exchanges freely between the accumulated nascent RNP and the surrounding nucleoplasm, and that it exits RNP with similar kinetics to its entrance. Overall, it appears that on transcription loops, nascent transcripts contribute to a dynamic self-organizing structure that exemplifies a phase-separated nuclear compartment.

**Electronic supplementary material:**

The online version of this article (10.1007/s00412-018-0667-8) contains supplementary material, which is available to authorized users.

## Introduction

Two fundamental aspects of nuclear organization have recently been substantiated using novel experimental approaches. One is that chromosome looping and loop structures at various levels underpin both the spatial organization of genomes in interphase nuclei and the establishment and regulation of gene transcription (Denker and de Laat 2016; Fudenberg et al. 2016; Hnisz et al. 2016; Rao et al. 2014). The other deals with the physical and functional organization of the interchromatin space, a prominent feature of which is the presence of a variety of nuclear bodies that are now thought to reflect the formation of liquid-liquid phase-separated compartments (Mao et al. 2011; Zhu and Brangwynne 2015). Indeed it has recently been suggested that both of these organizational principals could be in operation at sites of RNA polymerase II (pol II) transcription (Hnisz et al. 2017). However, many questions still remain about the formation and dynamics of individual loop structures and the detailed structure and organization of transcription sites in living cells. Fortunately, both these fundamental features of nuclear organization can be directly addressed by investigating the unusual lampbrush configuration that chromosomes adopt in the oocytes of animals such as amphibians that form large, yolky eggs (reviewed in Callan 1986; Gaginskaya et al. 2009; Gall 2014; Morgan 2002). (Even mammalian chromosomes, which as in other organisms that produce small eggs, do not exhibit a lampbrush configuration naturally, can be reprogrammed to adopt it simply by their being injected into amphibian oocytes (Liu and Gall 2012)).

Lampbrush chromosomes are typically seen as de-condensed diplotene bivalents from which extend thousands of DNA loops that are highly transcribed by pol II and are visible by standard light microscopy. These transcription loops, which range from tens to hundreds of kilobases of DNA depending on the species, project from more compact and transcriptionally inert chromatin domains termed “chromomeres.” Moreover, individual transcription units can themselves be resolved on the loops; this is because nascent transcripts are so densely packed, with their transcription complexes being spaced only about 100 bp apart that a visible ribonucleoprotein (RNP) “matrix” is formed around the transcribed DNA. This high density of nascent transcripts is reflected in a rate of steady-state nuclear RNA synthesis by pol II that is about a thousand-fold higher in oocytes than in typical somatic cells (Anderson and Smith 1978; Davidson 1986). Indeed, lampbrush chromosomes provide the first, classic case of what is now recognized as “hypertranscription” (Percharde et al. 2017).

The ability to analyze in real time individually resolvable loops representing specific DNA loci is beyond the approaches of microscopy and proximity ligation used currently to study interphase chromatin loops (Bystricky 2015; Denker and de Laat 2016). Therefore, analyses of “live” lampbrush transcription loops could offer novel insights into structural and temporal dynamics of chromatin loops per se. Moreover, since each loop represents an individual pol II transcription site, they could greatly inform our understanding of the architecture and physical form of such sites in interphase nuclei, sometimes known as “transcription factories,” which are much more challenging to visualize directly (Papantonis and Cook 2013; Rickman and Bickmore 2013; Sutherland and Bickmore 2009; Weipoltshammer and Schofer 2016). However, our current understanding of lampbrush chromosomes has been obtained from spread preparations in which chromosomes are isolated from the nucleus in a non-functional state and detailed observation of individual transcription loops over time in living oocytes has yet to be achieved. An underlying practical problem in studying live oocytes is that the accumulation of pigment and yolk granules obscures from view the nucleus and the structures within it. However, nuclei that have been hand-isolated from amphibian oocytes into mineral oil have been shown to retain the characteristics of functional nuclei and have been used successfully to study aspects of nuclear physiology (Paine et al. 1992). More recently, protein dynamics of chromatin and nuclear bodies have also been determined in oil-isolated nuclei (Austin et al. 2009; Handwerger et al. 2003; Nizami and Gall 2012). Crucially also, intact lampbrush chromosomes and their loops can be detected by standard DIC microscopy in isolated nuclei, although the inherently low levels of contrast in the images limit detailed analysis of transcription loops (Patel et al. 2008).

In order to use this system to provide a live-imaging approach for analyzing the structure and dynamics of transcription loops and their transcription units over time, a means of marking individual loops with a fluorescent label was required. A variety of macromolecules such as snRNPs and hnRNPs (Pinol-Roma et al. 1989; Wu et al. 1991) have previously been identified as components of the nascent transcript RNP of either many or just a subset of loops (Bellini et al. 1993; Eckmann and Jantsch 1999; Jantsch and Gall 1992; Morgan 2007; Roth and Gall 1989). Here, the selective targeting to nascent RNP of fluorescent fusions of the multifunctional RNA-binding protein, CELF1 (Barreau et al. 2006), was exploited to label individual loops in intact *Xenopus* oocyte nuclei. This enabled loops to be imaged in real time and also allowed the dynamic flux of CELF1 in morphologically defined pol II transcription units to be measured using photophysical approaches. The latter provides a means to test whether loop nascent transcripts inhabit a genuine nuclear compartment analogous to classic nuclear bodies (Mao et al. 2011).

Two important features of transcription loops are described here. First, observations of individual loops in real time in single functional nucleus revealed a range of lifetimes ranging from loops that persisted over hour-long observation periods to those that were unstable and shrank markedly over shorter time frames. Moreover, loop stability appeared to be correlated with the presence of nascent RNP. Secondly, the nascent RNP component of transcription loops exhibited a dynamic behavior that suggests that active pol II transcription units do comprise self-organizing structures that exemplify phase-separated nuclear compartments. Overall, these observations of lampbrush chromosome transcription loops underline a crucial role for nascent RNP in determining the structural dynamics of chromosome loops, which may have implications for transcription sites more generally.

## Materials and methods

### Expression of fluorescent protein fusions

The coding region of human CELF1 (CUG-BP) obtained from a *myc*-tagged construct (Morgan 2007) by PCR was re-cloned between an upstream T3 RNA polymerase promoter and a downstream fluorescent protein ORF that had two hemagglutinin (HA) repeats encoded at its C-terminus. For photoactivatable GFP derivatives, the coding region from vector pPA-GFP-N1 (Patterson and Lippincott-Schwartz 2002) was used. Constructs encoding fluorescent U1snRNP C protein were made by replacing the CELF1 coding region with the *Xenopus* U1C coding region produced by PCR from plasmid pCMA (Jantsch and Gall 1992). Constructs encoding fluorescent coilin fusions for the experiments shown in Online Resource 1 were made using a *Xenopus* coilin coding region produced by PCR from plasmid PAGFP-Xcoil-HA (Deryusheva and Gall 2004). Capped, sense-strand transcripts were prepared using a T3 RNA polymerase mMessage mMachine Kit (Ambion). Of each transcript, 2–20 ng was injected in a constant volume of 4 nl into the cytoplasm of defolliculated stage IV-V *Xenopus laevis* oocytes (European Xenopus Resource Centre, Portsmouth, UK) using a PLI-100 Pico-injector (Medical Systems Corp.), followed by incubation at 19 °C for 20–48 h.

### Preparation and immunostaining of nuclear spreads

Nuclear spreads were prepared from oocyte nuclei that had been manually dissected in isolation medium (83 mM KCl, 17 mM NaCl, 6.5 mM Na_2_HPO_4_, 3.5 mM KH_2_PO_4_, 1 mM MgCl_2_, 1 mM DTT, pH 6.9–7.2). Spread preparations were made using the procedure developed by Gall (Gall and Wu 2010), except that for unfixed preparations, the dispersal chambers were constructed with a coverslip rather than a microscope slide forming the floor of the chamber. For fixed preparations, slide-based chambers were used and the spreads were fixed for a minimum of 15 min and a maximum of 2 h in 2% paraformaldehyde made up in phosphate-buffered saline (PBS; 137 mM NaCl, 2.7 mM KCl, 10.2 mM Na_2_HPO_4_, 1.8 mM KH_2_PO_4_, pH 7.4) containing 1 mM MgCl_2_.

Prior to staining with primary antibodies, fixed preparations were rinsed in PBS and blocked by incubation in 10% fetal calf serum in PBS for 30 min. The spreads were then incubated for 1 h at room temperature with primary antibodies, rinsed briefly with 10% fetal calf serum, and then incubated for 1 h with secondary antibodies diluted in PBS. Preparations were stained with DAPI (0.5 μg/ml in PBS) and mounted in 50% glycerol/PBS. Primary antibodies diluted in 10% fetal calf serum as necessary were α-pol II (mAb H5 (Warren et al. 1992)) culture supernatant, α-BrdU (mAb BMC 9318; Roche) 2 μg/ml, α-CUG-BP1/hCELF1 (mAb 3B1; Abcam) 1:500 dilution, α-pol I/III (mAb No34; a gift from Marion Schmidt-Zachmann) 1:500 dilution, and α-HA (mAb 3F10; Roche) 0.5 μg/ml. Secondary antibodies, used at dilutions of 1–5 μg/ml, were Alexa 488-conjugated goat anti-mouse IgG or goat anti-rat IgG and Alexa 594-conjugated goat anti-mouse IgM or goat anti-mouse IgG (all Molecular Probes).

### Isolation of oocyte nuclei in oil

The procedure for isolating intact oocyte nuclei under mineral oil (Sigma) followed that initially devised by Paine et al. (1992) but using the modified observation chambers described by Patel et al. (2008), which are needed to preserve the integrity of lampbrush chromosomes. Unless prior extended incubation under oil was required, nuclei extruded from oocytes into oil were immediately transferred to observation chambers and examined by fluorescence microscopy within 10–20 min. Nuclear isolation and incubations were carried out at 18–20 °C. RNA synthesis in oil-isolated nuclei was detected by injection of 1.3 nl of 27 mM BrUTP (i.e., 35 pmol), which is in the range estimated for the endogenous nuclear UTP pool (Paine et al. 1992; Woodland and Pestell 1972). To detect nuclear RNA synthesis in intact oocytes, 4 nl of 80 mM BrUTP (Sigma) was injected into the cytoplasm to produce a similar nuclear BrUTP concentration to that achieved in the direct nuclear injections. Incorporation of BrUTP was assayed by immunostaining aqueous spreads prepared from oil-isolated nuclei using the technique devised by Gall and described in Patel et al. (2008).

### Microscopy and photokinetic experiments

Wide-field imaging was performed with an Olympus BX-60 microscope and Princeton Instruments digital CCD camera (Roper Scientific). FRAP and photoactivation experiments were performed with a Zeiss LSM880 laser scanning confocal microscope using a ×63, NA 1.4 oil immersion objective. Fluorescent images of mCherry-labeled loop loci were collected as single optical sections of 1–2 μm using the 561-nm laser line at 0.3–1% intensity. For FRAP, loci were bleached by scanning the 561-nm laser beam at full intensity 10–20 times over a region of interest (ROI) containing a whole locus. For photoactivation experiments, suitable loci double-labeled with mCherry and PA-GFP were found and imaged with the 561-nm laser allowing an ROI containing the locus to be defined. Then, either the whole locus was photoactivated by scanning a 405-nm laser beam at 10% intensity 5–10 times over the ROI or a sub-region of the locus was photoactivated using a diffraction-limited spot produced by targeting the 405-nm laser beam to a single pixel within the ROI. Immediately prior to and after photoactivation, images were collected with a 488-nm laser line attenuated to 0.5% intensity. Photokinetic measurements of CELF1 in transcription loops were compared to the dynamics of co-expressed mCherry- and PA-GFP-tagged coilins in the histone locus bodies (HLBs) of oil-isolated nuclei. Controls for the effects of photobleaching during imaging employed either fixed or unfixed nuclear spread preparations mounted in saline. Oil-isolated nuclei and spread preparations were imaged at 18–20 °C.

Images exported as TIFF files were analyzed with iVision-Mac (BioVision Technologies) and graphs plotted with Microsoft Excel. Mean pixel intensity values were normalized after background subtraction with respect either to pre-bleach and immediate post-bleach fluorescence values or to pre-activation and immediate post-activation values for FRAP and photoactivation experiments, respectively (Rino et al. 2014).

## Results

### Fluorescent protein fusions are correctly targeted to nascent transcripts of lampbrush chromosome loops

**Fig. 1:**
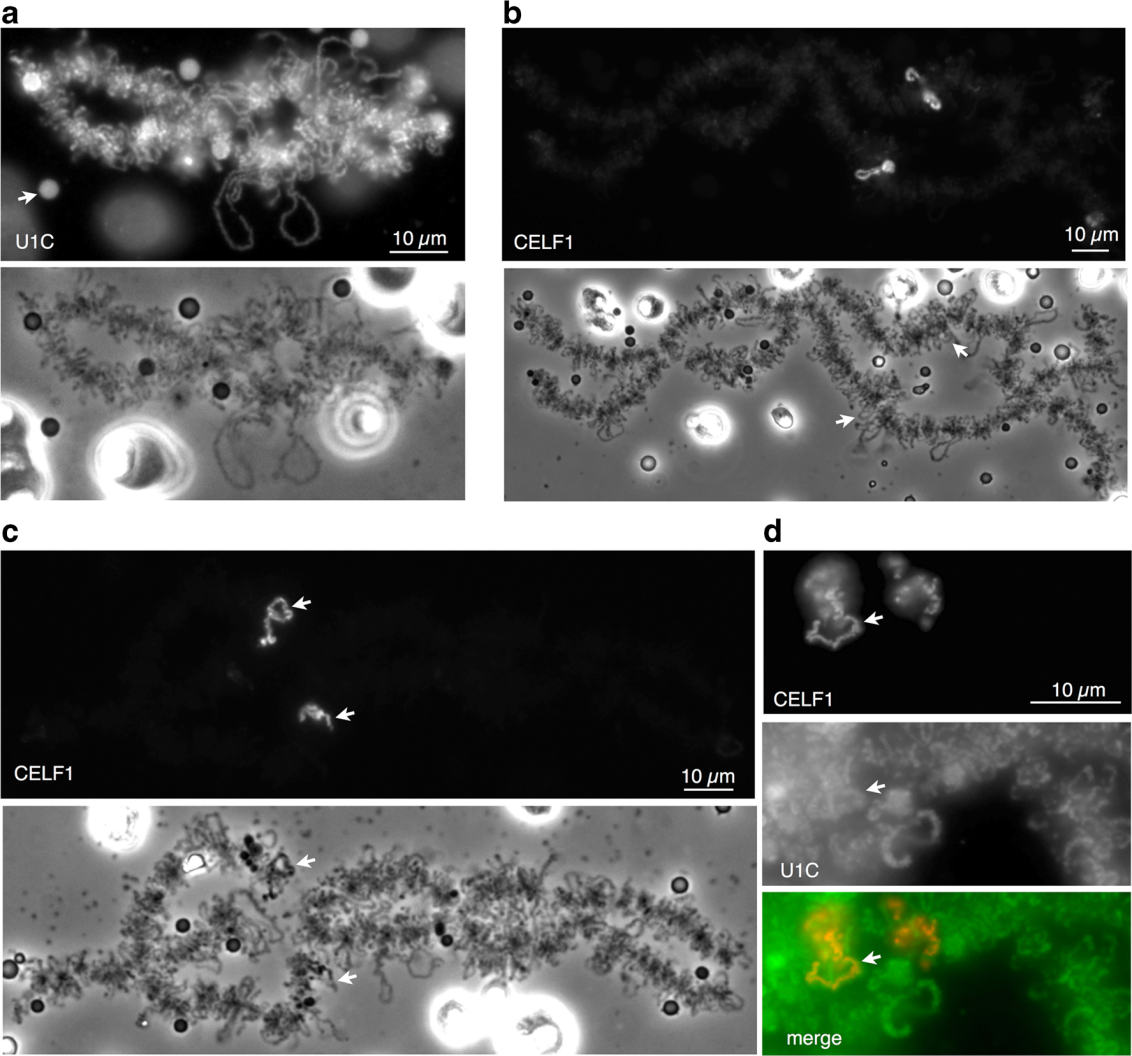
Distinctive chromosomal targeting of fluorescently tagged RNA-binding proteins in unfixed nuclear spread preparations. Fluorescence and phase contrast images showing **a** U1C.mCherry targeting to loops of a lampbrush bivalent and to a type of nuclear body, the B snurposome (arrow). The large, highly refractile nuclear bodies in the phase contrast image are extrachromosomal nucleoli. **b** Specific CELF1.GFP targeting to four lateral loops, which correspond to the four chromatids comprising each locus in the 4C lampbrush bivalent; two of these morphologically unremarkable loops at homologous sites are arrowed in the phase contrast image (note that one of the lower pairs of sister loops is collapsed onto the chromosome axis and is viewed “end-on”). **c** A set of loops with a distinctive contorted morphology are targeted by CELF1.GFP, contorted loops at homologous loci (arrows) on LBC 7 in an unfixed spread preparation. Note that only the phase-dark regions of the loops appear highly fluorescent (lower arrow). In **d**, co-expression and co-targeting of U1C.mCherry (pseudocolored green in merge) and CELF1.GFP (pseudocolored red) to contorted loops are shown. One of the two homologous contorted loop loci is arrowed in each panel

In order to determine whether nascent RNP-binding proteins could be used to label loops in “living” nuclei, it was necessary first to test that fluorescent fusions of candidate proteins would be efficiently expressed and specifically targeted in *Xenopus* oocytes. Synthetic transcripts encoding the snRNP C or CELF1 splicing factors fused to mCherry or GFP were injected into the oocyte cytoplasm. After incubation for 1 to 2 days, nuclei were dissected from oocytes and their nuclear envelopes were removed manually. The nuclear contents were allowed to disperse in saline and to settle onto a coverslip that formed the base of an observation chamber. This relatively rapid approach provides good morphological preservation of spread lampbrush chromosomes that are unfixed but non-functional. For both the fluorescent fusions, targeting was readily monitored in a chromosomal context by wide-field fluorescence microscopy. In newt oocytes, the U1 snRNP C protein has previously been shown to be targeted to the RNP of most lampbrush chromosome loops and to numerous extrachromosomal bodies (B snurposomes) that are thought to be the oocyte equivalent of splicing speckles (Gall et al. 2004; Jantsch and Gall 1992). A U1C.mCherry fusion showed the same targeting pattern in *Xenopus* oocytes (Fig. 1a). In contrast to the widespread distribution pattern of this general splicing factor, the multifunctional regulator CELF1 has been shown previously to have a much more restricted distribution, being confined to the nascent RNP of a small number of specific loops and sometimes even to just a sub-region of a loop (Kaufmann et al. 2012; Morgan 2007). The CELF1.GFP and mCherry fusions also showed this far more restricted distribution and were specifically targeted only to the loop RNP of several loci within each *Xenopus* LBC set (Fig. 1b). Moreover, the specific targeting behaviors of CELF1.GFP and U1C.mCherry were recapitulated in oocytes injected with a 1:1 mixture of both transcripts in order to co-express the proteins (Fig. 1d).

Whereas the nascent RNP compartments of most of the loops targeted by fluorescent CELF1 were morphologically unremarkable (Fig. 1b), those at one locus, which were repeatedly the brightest fluorescent structures in nuclear spreads, did possess a distinctive appearance (see Fig. 1c, d). Although clearly loop-derived, the RNP matrix was bulkier than that of typical loops and appeared dark and highly contorted in phase contrast and often suggestive of intra- and even inter-sister fusion of the loop RNP. It was often difficult therefore to follow a clear loop-like track throughout the length of these contorted loops, which are examples of a class of morphologically distinctive or “marker” loops, so-called because they are repeatable and often species-specific features that can be used for chromosome identification purposes (Callan 1986). CELF1 appeared to be targeted rapidly and efficiently to the contorted loops, which were the only detectable fluorescent structures seen after short incubations of 3–4 h. CELF1 often appeared to be confined predominantly to sub-regions of the contorted loops (Fig. 1c), and this contributed to a marked variation in fluorescent images of different examples of the contorted loops. Further characterization of the contorted loops mapped them to chromosome 7, showed that they were natural targets for endogenous CELF1 and confirmed that they were transcriptionally active structures (data shown in Online Resource 1).

### Functional transcription loops can be imaged in intact nuclei via CELF1 targeting

**Fig. 2:**
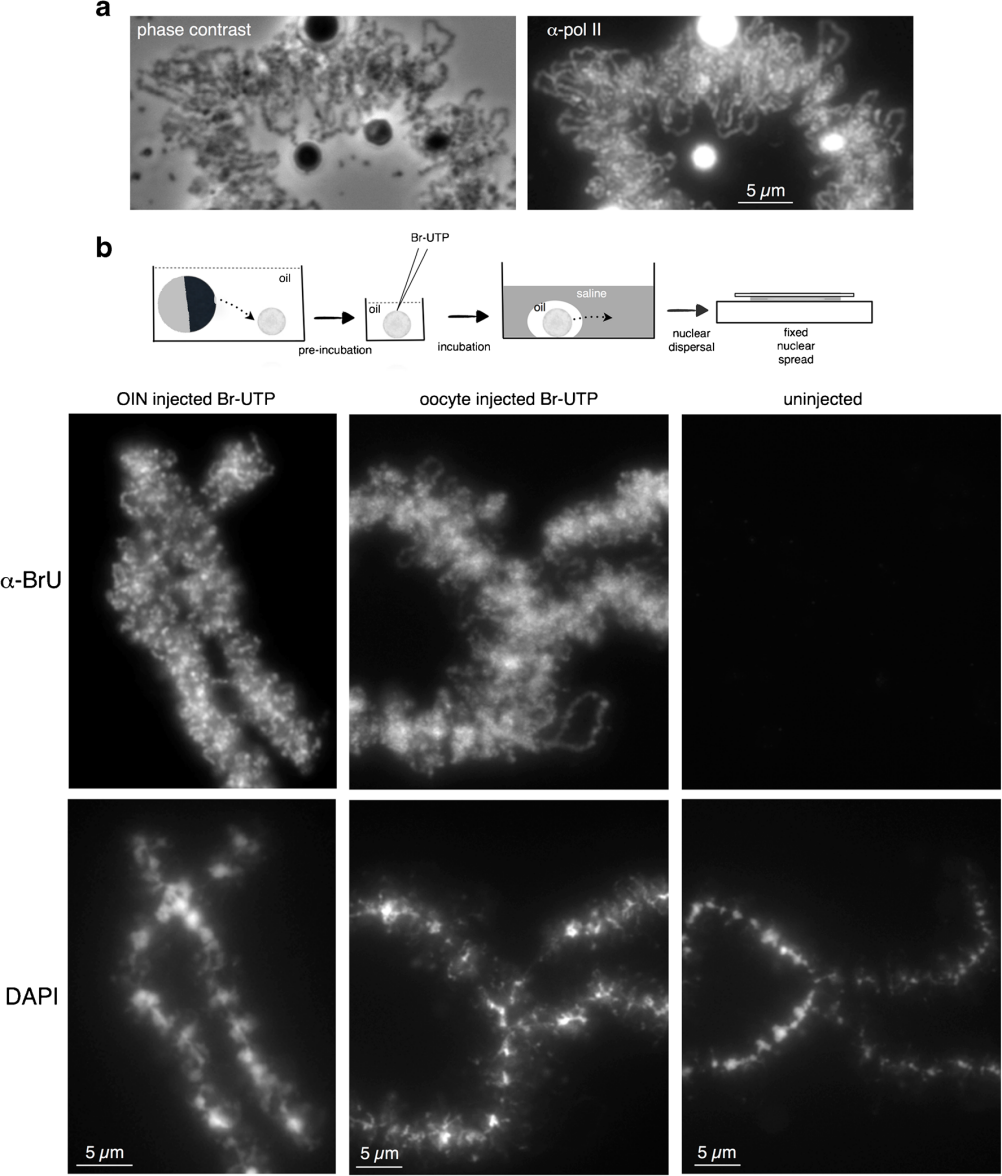
Retention of pol II transcriptional activity by transcription loops in oil-isolated nuclei. **a** Pol II immunostaining of lampbrush loops in a fixed spread prepared from an oocyte nucleus that had been isolated into oil and kept for about 3 h prior to spread preparation. The α-pol II monoclonal antibody that stains the loop axes recognizes a CTD phosphoisomer associated with transcriptionally active pol II. The brightly immunostained objects are B snurposomes, which contain epitopes that also cross-react with this antibody (Doyle et al. 2002). **b** Continued loop RNA synthesis in oil-isolated nuclei detected by Br-U incorporation followed by immunostaining. As summarized in the diagram, nuclei were isolated into oil and pre-incubated for about 2 h prior to injection of Br-UTP. Following incubation in oil for a further 3 h, each nucleus was transferred into a drop of oil under an aqueous solution into which it was then pushed, allowing the production of a fixed nuclear spread preparation. Immunostaining with an α-BrU antibody demonstrates that RNA synthesis was occurring on lampbrush loops at least 2 h after nuclear isolation (left-hand panels). As controls, a nuclear spread was prepared directly from an oocyte that had been injected 3 h previously with Br-UTP (center panels) and from an oil-isolated nucleus that was not injected with Br-UTP prior to incubation (right panels). Images are reproduced using the same contrast function

In order to determine if the targeting of fluorescent proteins to loop RNP in intact nuclei could provide a system to image functioning transcription loops rather than the non-functional ones analyzed above in nuclear spreads, it was necessary first to establish that pol II-directed synthesis of nascent transcripts continues on loops in these nuclei. The following two approaches were taken to address this: (1) isolated nuclei were incubated in oil for about 3 h and then recovered through saline in order to allow the production of standard fixed nuclear spread preparations. These preparations were then immunostained with a monoclonal antibody, mAb H5, that recognizes a pol II phosphoisomer associated with transcription elongation. The spread lampbrush loops showed intense immunostaining (Fig. 2a), indicating that transcriptionally competent pol II had at least stayed associated with loops during 3 h of nuclear incubation in oil. Next, in order to determine whether loop-associated pol II remained transcriptionally active after nuclear isolation, isolated nuclei were pre-incubated in oil for about 2 h and then directly injected with an amount of Br-UTP approximately equivalent to the endogenous nuclear pool. After 3 h of incubation in oil to allow Br-U to be incorporated into nascent RNA, the nuclei were recovered into saline and nuclear spreads prepared for immunostaining. Figure 2b shows that many loops of pre-incubated nuclei exhibit bright immunostaining for BrU. The intensity of immunostaining was comparable to that found in nuclear spreads made directly from intact oocytes that had been injected in the cytoplasm with Br-UTP and likewise incubated for 3 h (Fig. 2b). Taken together, these experiments show that pol II-directed transcription can continue on lampbrush loops for at least 2 h after nuclear isolation and the time course experiments described below were all undertaken within this period.

**Fig. 3:**
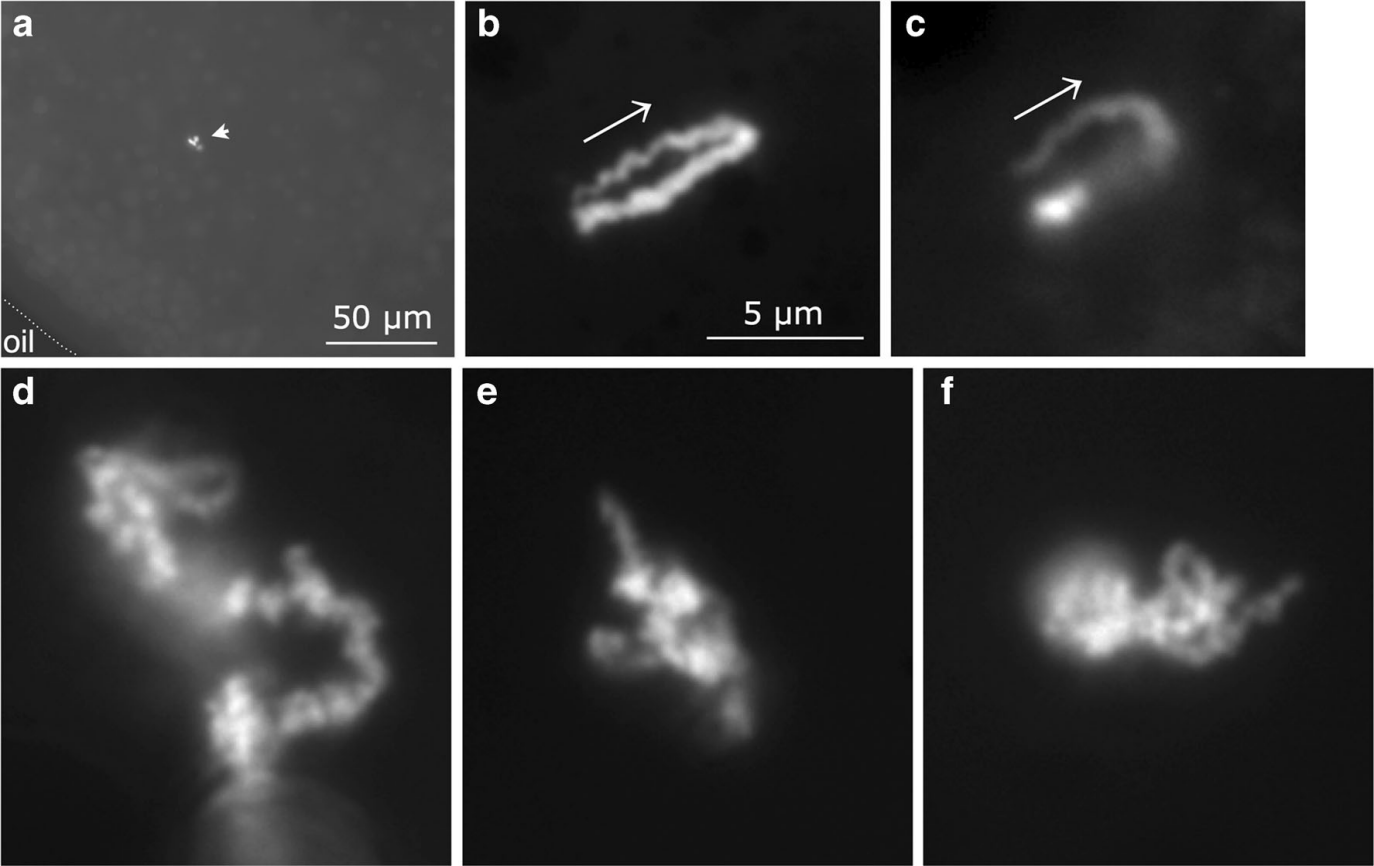
Transcription loops targeted by fluorescent CELF1 are detectable in intact, oil-isolated nuclei. **a** Survey view of part of an oil-isolated nucleus taken from an oocyte expressing CELF1.GFP. Brightly fluorescent structures (arrowhead) are detectable against a lower nucleoplasmic background fluorescence. Dotted line indicates position of oil/nuclear envelope interface. **b**–**f** Higher-magnification, wide-field images of lampbrush loops targeted by CELF1.mCherry in oil-isolated nuclei. Loops with either a typical, “thin-thick” morphology (**b**, **c**) or one characteristic of contorted loops (**d**–**f**) are shown. Arrows in **b**, **c** indicate the predicted direction in which pol II is tracking along these loops. Images **b**–**f** to same scale

**Fig. 4:**
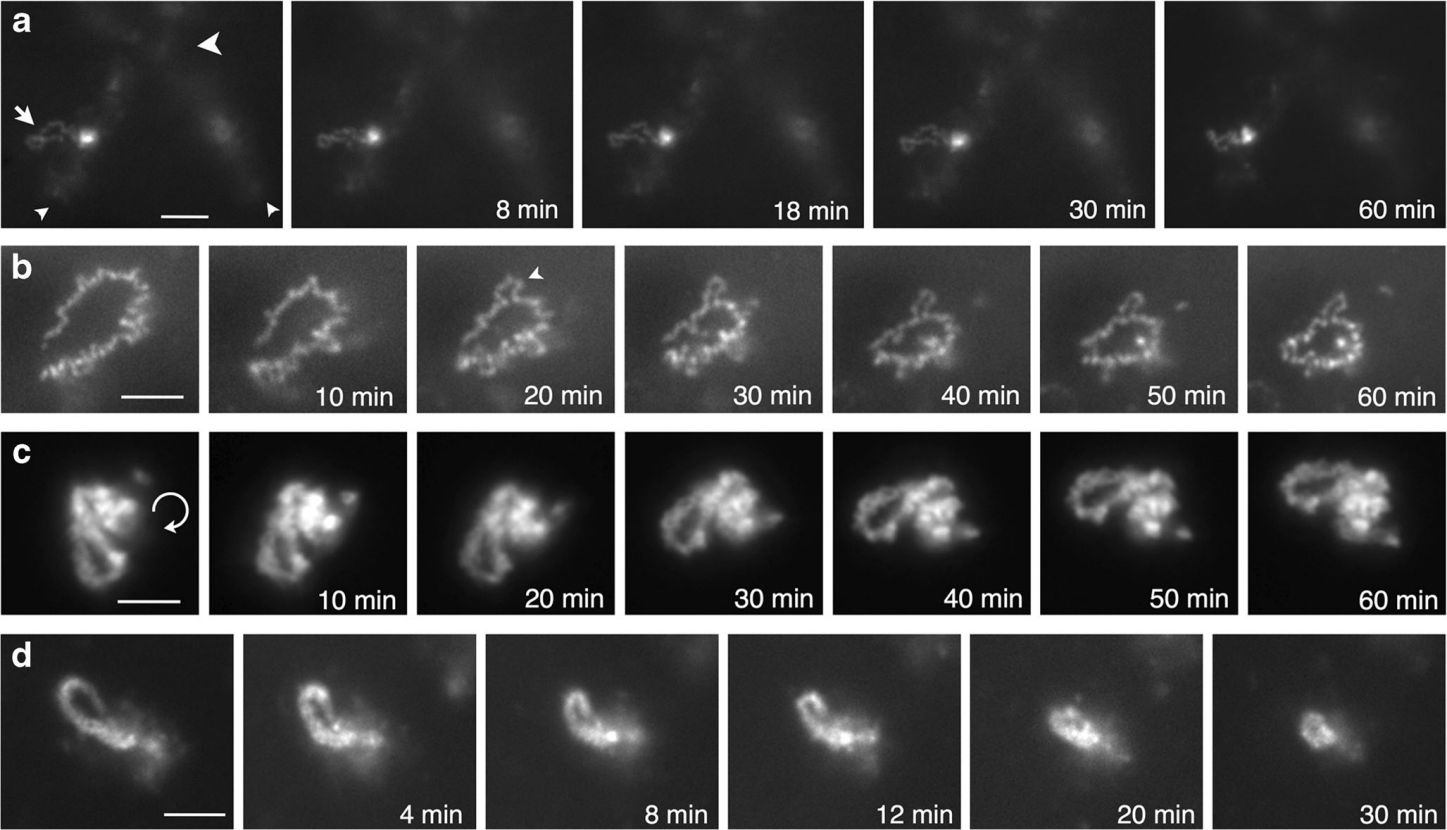
Stability of transcription loops over time in oil-isolated nuclei. **a**–**d** show images at regular time points of four transcription loops each targeted by CELF1-GFP and exhibiting a range of RNP compartment morphologies. **a** Survey view of a CELF1-targeted loop (arrow) extending from a lampbrush bivalent that is detectable via faint background labeling (location of a chiasma is indicted by a large arrowhead and approximate positions of the ends of the homologous chromosome arms are indicated by small arrowheads). The neighboring bright sister loop is viewed end-on and may be collapsed; the homologous locus in the homolog to the right is detectable in a different focal plane. The indicated loop appeared essentially unchanged over the time course. **b** Example of a loop that initially exhibits a convoluted/kinked morphology and which over time produces internal “sub-loops” (arrowhead). Although an essentially looped track with two definable insertions at its base is maintained over the time course, there also appears some contraction in overall loop length. **c** Example of a contorted loop locus that shows an orientation change (curved arrow) during the time course, although the complex morphology of the GFP-labeled RNP compartment appears stable over time. **d** Example of a loop that initially exhibits a typical “thin-thick” asymmetry in RNP distribution along its length. A marked reduction in loop axial length has begun by 8 min and by 30 min no longer are an overt loop-like form nor a clearly asymmetric distribution of RNP apparent. This time-lapse series was obtained from the same nucleus, and completed prior to, the one shown in **a**. All scale bars = 10 μm

It was also critical in attempting to image functioning loops that loop-targeted fluorescent protein fusions were detectable in intact nuclei over the fluorescence of untargeted protein fusions in the nucleoplasm. To determine this, the nuclei of oocytes co-expressing U1C.mCherry and CELF1.GFP were isolated under oil and then immediately examined by wide-field microscopy. In the former case, it was not possible to discern the presence of U1C protein in distinct nuclear structures against high nucleoplasmic levels of the protein. (The previous detection of loops via fluorescent U1C targeting to loop RNP in nuclear spread preparations (Fig. 1a) was likely possible because nucleoplasm is diluted about a thousand-fold when the nuclear contents are dispersed in saline.) However, even in the same oil-isolated nuclei in which U1C appeared evenly distributed, foci of bright CELF1 fluorescence were clearly detectable against a much lower nucleoplasmic background (Fig. 3a). From one to several discrete fluorescent structures were detectable per nucleus; these could sometimes be clearly seen in a chromosomal context in nuclei from oocytes, in which fluorescent CELF1 expression was sufficiently high that lampbrush bivalent axes were discernible due to a weak fluorescence brought about by the non-specific association of CELF1 (Fig. 4a).

The brightly labeled structures of intact nuclei exhibited the same range of morphologies found in loops and loop-related structures in spread preparations, although without the flattening effects inherent in spread preparations, individual loops (and their sisters or homologs) typically fell in multiple focal planes. The most commonly observed and most highly fluorescent structures labeled in intact nuclei had all of the general morphological characteristics noted above for spread contorted loops. Although, as in spreads, individual examples varied widely in appearance (Fig. 3d–f), they were usually large and exhibited a complex, contorted shape, often with CELF1 targeting seeming to involve only part of a loop. However, in some oil-isolated nuclei, several additional fluorescent loci were detectable that resulted from the targeting of CELF1 to loops with the simpler morphology typical of most loops in traditional aqueous spreads of *Xenopus* LBCs. These loops were about 10–20 μm in length and were not extensively fused like the contorted loops but extended into the nucleoplasm and followed a fairly linear and clearly loop-like track with apparent insertion points on the chromosome axis (Fig. 3b, c). The general resemblance between loops in aqueous spreads and those in intact, oil-isolated nuclei using DIC microscopy has previously been noted (Patel et al. 2008). However, the greater contrast available in these fluorescent images provides more detail of the nascent transcript compartment of typical transcription loops. In particular, some provided clear examples of a gradual increase in the width of the CELF1-targeted RNP matrix along the length of the loop (Fig. 3b, c). This classic morphological feature of lampbrush loop transcription units arises from the gradually increasing mass of adjacent transcripts in a tightly packed array of nascent RNPs undergoing unidirectional transcription elongation (Callan 1986). The “thin-to-thick” asymmetry reveals the polarity of ongoing transcription in these functioning transcription units and therefore the direction in which the pol II array is tracking along the static loop DNA (Fig. 3b, c).

### Stability of transcription loops

In addition to providing morphological details of the RNP compartments of targeted transcription units, CELF1 fluorescence enabled real-time observation of transcription loops. Over time courses of up to an hour, two broad types of dynamic behavior were observed at about equal frequency. Among transcription loops exhibiting a simple RNP matrix morphology, some maintained an extended and clearly loop-like track over the time course without substantial changes in overall length or in the appearance of the nascent RNP component (Fig. 4a, b). However, these “long-lived” loops could exhibit subtle changes in appearance over time due to an apparent flexibility in a loop’s precise axial track in 3D and from changes in focal plane resulting from motion of the whole loop. Similarly, a stable appearance was also the case for the contorted loops, although here, recognition of the complete track of the underlying chromatin loop was usually not possible because the complex RNP matrix rather than the underlying loop DNP axis is the dominant determinant of the overall loop shape. However, examples from different nuclei of contorted loop loci intensely labeled by fluorescent CELF1 fusions were observed over extended periods (Fig. 4c), sometimes as single focal planes by confocal microscopy (Fig. 6a). Again, the size and complex shape of the RNP compartment of each contorted loop locus remained broadly similar over the course of an hour but most underwent modest changes in orientation or in appearance due to conformational changes.

In contrast to those exhibiting a stable loop morphology, some simple loops showed a marked reduction in loop length and in the overall amount of associated fluorescent RNP during periods as short as 20–30 min (Fig. 4d); these changes were accompanied by the loss of an overtly loop-like shape and in some cases, the virtual disappearance of the loop and its fluorescent RNP. Such “short-lived” loops could be observed prior to extended observations of long-lived loops in the same nucleus (Fig. 4a vs. d), suggesting that they were not a result simply of total nuclear dysfunction.

### Dynamics of loop nascent RNP

**Fig. 5:**
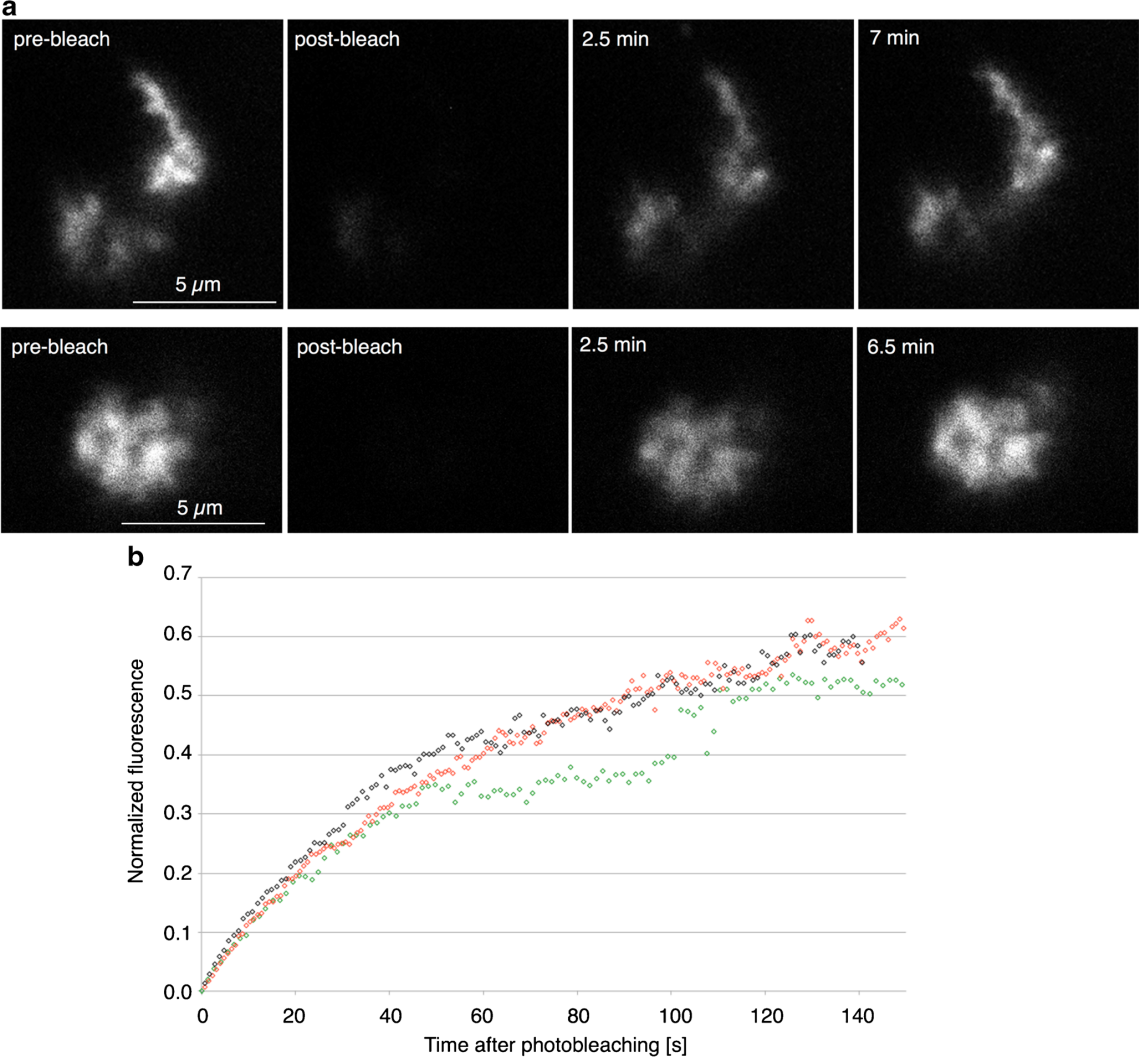
FRAP of CELF1.mCherry in contorted loop loci of oil-isolated nuclei. **a** Selected images from two confocal FRAP time courses. Photobleaching was performed on individual contorted loop loci using a bleach region that completely enclosed each structure. The recovery of 80 to 100% CELF1.mCherry fluorescence in single optical sections of 2 μm over time is shown. **b** FRAP recovery curves based on three contorted loop loci of varying size and appearance. Because of their morphological differences, individual FRAP curves for the different loci were not averaged but all predict similar half-times for recovery of 1.5 to 2 min

A further aspect of the dynamic properties of transcription loops can be investigated using the targeting of fluorescent CELF1 to their nascent RNP. This concerns the extent to which a transcription unit can be considered a phase-separated nuclear compartment. An important general property of nuclear compartments in addition to structural stability is their existence at a dynamic steady state as exemplified by the constant exchange of components with the surrounding nucleoplasm (Dundr and Misteli 2010; Mao et al. 2011). Here, fluorescence recovery after photobleaching (FRAP) of CELF1.mCherry in oil-isolated nuclei was carried out to determine if there was dynamic entry of CELF1 from the nucleoplasm into the nascent RNP matrix of contorted loops. These loops were used preferentially because of their reliable occurrence and identification, bright labeling, and a relative lack of mobility coupled with large target size. Photobleaching was performed on individual contorted loop loci using a bleach region that completely enclosed each structure. Recovery of CELF1.mCherry fluorescence was quantified from images collected as single optical sections for periods during which major alterations in shape or position did not affect the reliability of measurement. In the FRAP experiments shown in Fig. 5a, individual loci were observed for 6–7 min, by which time 80 to 100% fluorescence recovery had occurred. Recovery curves (Fig. 5b) were plotted based on acquiring images at intervals of about a second for 2.5–3 min for three contorted loop loci of varying size and appearance. Because of their morphological differences, individual FRAP curves for the different loci were not averaged but they all predict similar half-times for recovery of 1.5 to 2 min. In contrast, no recovery was seen after bleaching fluorescent loci in fixed nuclear spread preparations (data not shown). To provide a comparison for CELF1 behavior, FRAP experiments using the approach of Handwerger et al. (2003) to measure coilin dynamics in the HLBs of oil-isolated nuclei were carried out. Coilin.mCherry fluorescence recoveries were similar to those described in earlier studies of coilin.GFP (Deryusheva and Gall 2004; Handwerger et al. 2003) and were noticeably slower than those found for CELF1. The half-time for recovery of a bleached spot within the HLB shown in the supplementary data (Online Resource 1) was about seven times longer than those measured for CELF1.mCherry in contorted loops.

**Fig. 6:**
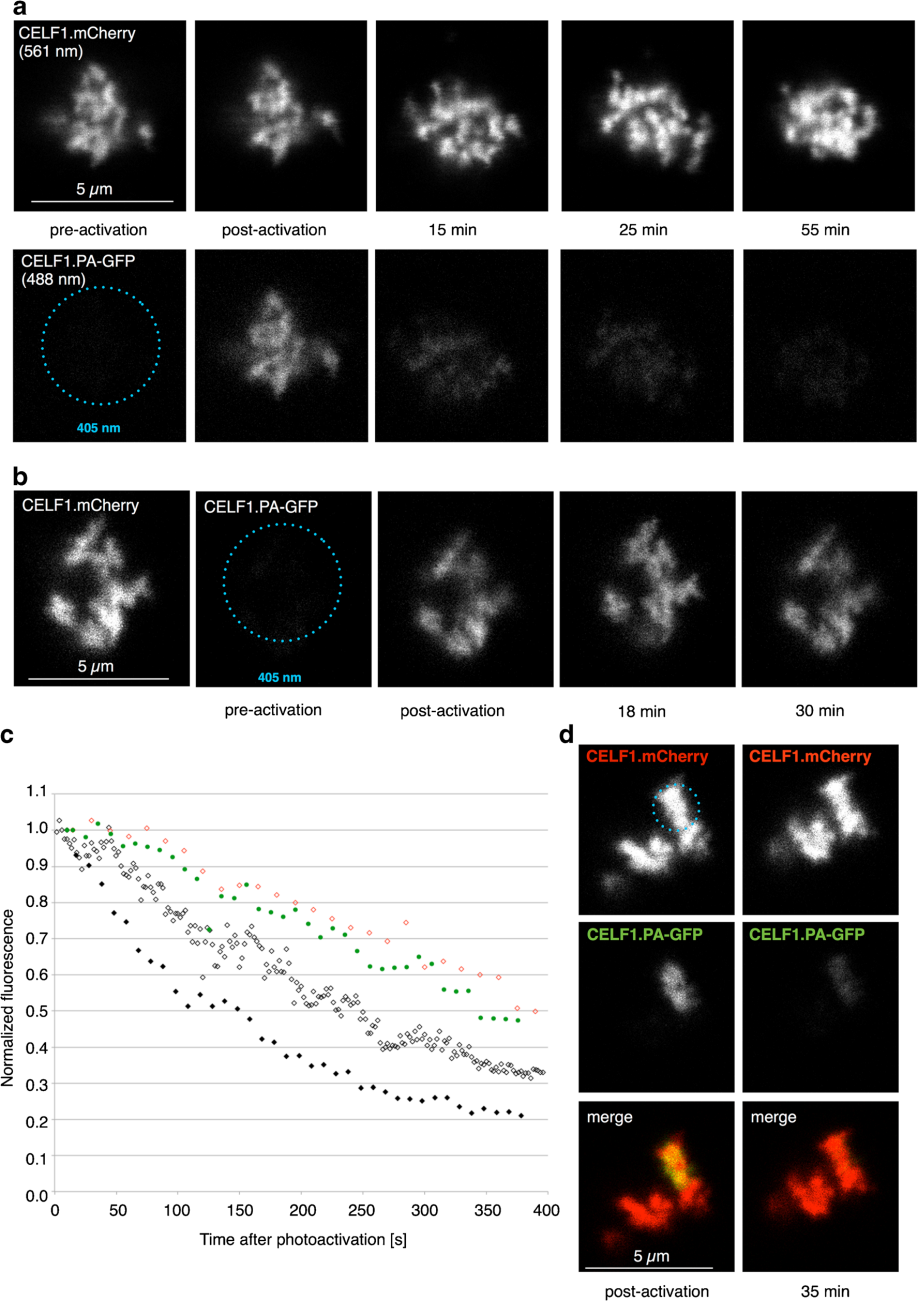
Photoactivation of CELF1.PA-GFP in contorted loop loci and fluorescence loss over time. **a** Contorted loops from an oil-isolated nucleus that contain both CELF1.mCherry, which is detected at 561 nm in single optical sections, and unactivated CELF1.PA-GFP, which is not initially detectable at 488 nm. After photoactivation at 405 nm within an ROI encompassing the whole locus (dotted circle), bright fluorescence at 488 nm is detectable. The intensity of fluorescence detected at 488 nm in the immediately post-activation image becomes reduced over time until it is undetectable. The overall fluorescence of CELF1.mCherry in the same loops appears unaltered, although the locus undergoes conformational changes over the time course. **b** Experiment as in **a** except using a spread preparation in which the contorted loops are suspended in saline rather than nucleoplasm. Again, robust photoactivation of CELF1.PA-GFP was detected at 488 nm in contorted loop loci but, although imaged under the same conditions used for **a**, there was no appreciable loss of fluorescence relative to immediately post-activation levels. **c** Fluorescence decay curves plotted separately from quantitative data obtained for four different contorted loop loci. These show that 50% of the initial fluorescence intensity of CELF1.PA-GFP are lost within 2.5–6 min of photoactivation. Three of the curves (red and black symbols) were obtained from loop loci that were photoactivated in their entirety, while one (green symbols) is derived from the experiment shown in **d**, in which only a sub-region of a contorted loop locus was photoactivated. **d** Regional photoactivation of CELF1.PA-GFP in a contorted loop locus was obtained by confining the 405-nm laser beam to a diffraction limited spot targeted by reference to the co-localized CELF1.mCherry image (dotted circle). Loss of fluorescence at 488 nm from the photoactivated region occurred over time at similar rates to the experiment shown in **a**

In order to gain further insights into the dynamic behavior of CELF1, an approach using a photoactivatable GFP (PA-GFP) was developed. In principle, if a CELF1/PA-GFP fusion could be photoactivated in the RNP matrix of contorted loops, then the decay of fluorescence would enable any exit of CELF1 from the loop to be detected. The prior identification of potential CELF1-targeted loops before photoactivation was achieved by co-expressing CELF1-mCherry and PA-GFP-fused CELF1. After locating contorted loop loci via their mCherry fluorescence, a region of interest enclosing a whole locus was defined from the image. Co-targeted CELF1.PA-GFP was then subjected to photoactivation by scanning a 405-nm laser beam over the region. Pre-activation and immediately post-activation images of single optical sections were obtained simultaneously at 561 nm (for mCherry) and 488 nm (for activated GFP) and at intervals thereafter to assess the extent of fluorescence decay. It can be seen from the typical images shown in Fig. 6a that PA-GFP fluorescence was indeed activated throughout the targeted contorted loops and that the mCherry and PA-GFP labeling patterns closely matched each other. However, whereas the CELF1.mCherry fluorescence remained relatively constant over periods of up to 1 h (Fig. 6a, upper panel), the activated fluorescence of CELF1.PA-GFP (Fig. 6a, lower panel) appeared to fade steadily over time and to be almost at pre-activation levels after 25 min. To ascertain whether this loss of fluorescence was related to photobleaching during imaging, photoactivation was repeated on spread preparations in which the nuclear structures are suspended in saline rather than nucleoplasm. Again, robust photoactivation of CELF1.PA-GFP was obtained in contorted loop loci but, although imaged under the same conditions as oil-isolated nuclei, there was no appreciable loss of fluorescence over time relative to immediately post-activation levels (Fig. 6b).

To estimate the rates of decay of CELF1.PAGFP fluorescence in contorted loops, single optical sections were imaged at 488 nm at regular intervals after photoactivation, except for periods when the loops underwent marked morphological changes. Fluorescence decay curves are plotted separately for three different contorted loop loci in Fig. 6c, and these show that 50% of the initial fluorescence intensity are lost within 2.5–6 min of photoactivation. Similar rates of PA-GFP fluorescence decay were estimated in examples in which only a sub-region of a contorted loop locus was photoactivated by confining the activating laser beam to a diffraction-limited spot (Fig. 6d). To provide a comparison with CELF1, the previous experiments of Deryusheva and Gall (2004) that examined the dynamics of photoactivated coilin in oil-isolated HLBs were repeated. The time taken for a 50% loss of the initial coilin.PA-GFP fluorescence from a photoactivated spot within an HLB was about 15 min (data shown in Online Resource 1), several times longer than that needed for 50% loss of CELF1.PA-GFP fluorescence from contorted loops. Overall, the results of FRAP and photoactivation experiments provide evidence for the rapid flux of a component in and out of the morphologically definable RNP matrix of transcription loops, a property that is indicative of a phase-separated nuclear compartment.

## Discussion

The approach described here involved targeting fluorescent fusions of the RNA-binding protein CELF1 to the nascent transcripts of functional lampbrush chromosomes suspended in the liquid nucleoplasm of intact oocyte nuclei. It has provided for the first time a means to image in real time the structure and dynamic behavior of individual transcriptionally active chromosome loops.

### Dynamic maintenance of individual transcription loops

About half of the transcription loops observed over periods of up to an hour remained recognizably loop-like during this period, while others underwent a marked shrinkage both in overall length and in the amount of the associated fluorescent nascent RNP. The persistence of an extended state found for long-lived loops (Fig. 4a–c) was correlated with their continuous coverage by nascent RNP, presumably due to the maintenance of hypertranscription by these loops. In contrast, a large number of previous investigations of lampbrush chromosomes suggest that the behavior of short-lived loops is a real-time demonstration of the effects of reduced nascent transcript coverage. For instance, exposure to transcriptional inhibitors results in the absence of extended lampbrush loops, whereas loops are re-extended when inhibitors are removed and transcription resumes (reviewed in Callan 1986; Patel et al. 2008). Moreover, global loop retraction also results from enzymatic digestion of nascent RNAs (Scheer et al. 1984), again suggesting that the degree of loop extension is affected directly by nascent transcript density. A biophysical explanation of the effect of RNP density on loop extension has been suggested from polymer modeling studies which show that the repulsive forces between closely packed nascent transcripts would be sufficient to straighten loops into an extended configuration (Marko and Siggia 1997). In this context, it should be noted that numerous general and loop-specific RNA packaging and processing components have been detected in the nascent RNP of transcription loops where they contribute to the formation of a hierarchy of nascent RNP particles (reviewed in Callan 1986; Morgan 2002). The instability of short-lived loops could be due to their sensitivity to the experimental manipulations, although this would have to be a feature of particular loops rather than a general one because long-lived loops that were essentially stable morphologically were observed in the same nuclei as short-lived ones. Alternatively, the shrinkage and disappearance of certain loops might be the result of a programmed or stochastic variation in the lengths of time that different loops are able to maintain hypertranscription, perhaps akin to transcriptional bursting (Coulon et al. 2013).

Interestingly, the apparent requirement for a continuous active process to maintain transcription loops in an extended configuration is also a feature of emerging models for the creation and maintenance of other types of chromosome loop structure. These loops have been revealed by recent studies that primarily use mammalian interphase nuclei and a variety of imaging, proximity ligation, and modeling approaches (reviewed in Denker and de Laat 2016). Such studies have suggested the existence of loop-like structural units at a variety of length scales that have been variously described as sub-TAD loops, insulated neighborhoods, enhancer-promoter loops, loop domains, and CTCF-contact domains (Hnisz et al. 2016; Phillips-Cremins et al. 2013; Rao et al. 2014; Tang et al. 2015). Some of these loop types could actually overlap (Hnisz et al. 2016), and simply on the basis of length alone, it seems possible that the smaller types of interphase loop, which measure around a hundred kilobases (Rao et al. 2014), are equivalent in some respects to lampbrush transcription loops: the typical *Xenopus* loops seen here averaged 10–20 μm in length and, given the absence of nucleosomal packaging in loops at these maximal transcription rates (Scheer 1978), each would comprise 30–60 kb of B-conformation DNA. Direct observation of individual interphase loops and an understanding of their in vivo dynamics are currently unavailable, but it has been suggested from computational polymer modeling that individual loops will exhibit sporadic and stochastic appearance in populations of living cells (Dekker and Mirny 2016). In turn, it has been suggested that the presence of an individual loop at a given instant in a given cell could depend on continuous activity by topological machines driving a dynamic process of “extrusion” (Fudenberg et al. 2016; Goloborodko et al. 2016). A need for some kind of continuous active process for loop extrusion has an obvious parallel to the dynamic interrelationship of pol II transcription and loop extension in lampbrush loops discussed above. Indeed, roles for pol II in interphase loop extrusion have been suggested, either by its acting directly as a static motor protein exerting traction on loop DNA (Dekker and Mirny 2016; Lee et al. 2015; Papantonis and Cook 2013) or by mobile pol II complexes shunting other molecular machines such as cohesin along a loop during divergent, bidirectional transcription (Busslinger et al. 2017). However, what appears a distinctive feature of the pol II-dependent extension of lampbrush loops is the dominant structural role played by the unidirectional accumulation of nascent RNP particles.

### Transcription units as dynamic nuclear compartments

A further property of the loop RNP in intact nuclei was revealed here by analyses of contorted loops, an unusual set of loops in which the transcription units exhibit a morphologically highly complex nascent RNP matrix. The contorted loops were the most readily and repeatedly identified labeled loops in isolated nuclei and permitted photokinetic approaches to investigate the dynamics of CELF1 interactions with nascent RNP. In FRAP experiments, the fluorescence associated with CELF1 recruitment by the contorted loop RNP recovered with a half-time of about 2 min after bleaching the entire structure. The simplest explanation of the fluorescence recovery is that it results from the equal exchange of bleached CELF1 associated with nascent transcripts for unbleached CELF1 from the nucleoplasm. Similarly, in photoactivation experiments, activated fluorescence was lost from contorted loop RNP with half-times of only a few minutes, which by contrast was not found when the loops were isolated into saline. Again, a straightforward interpretation is that exit results primarily from the progressive exchange of activated CELF1.PA-GFP associated with RNP throughout the loop for equal amounts of unactivated CELF1 from the nucleoplasm. Overall, it appears that CELF1 exits the contorted loop RNP matrix with kinetics similar to those with which it enters and this also underlines the continuing functional activity of transcription loops in intact, isolated nuclei. Moreover, extended observation of individual contorted loops showed them to be stable morphologically over time and that, given the dynamic flux of CELF1, the RNP matrix must maintain its structural integrity at a steady state rather than simply reflecting molecular aggregation or co-localization. In these crucial respects, the nascent RNP matrix of transcription loops exhibits the dynamic properties that are the defining feature of nuclear compartments generally (Dundr 2012; Mao et al. 2011).

Since lampbrush chromosomes exhibit thousands of lampbrush loops each surrounded by a visible RNP matrix, the formation of a nuclear compartment would appear to be a common property of hyperactive pol II transcription units. As suggested for nuclear compartments in general, compartmentalization involving nascent pre-mRNP would potentially increase the rate, efficiency, and fidelity of processes occurring on these transcripts such as spliceosome assembly and co-transcriptional splicing, 3′ end processing, and hnRNP assembly. The existence of such compartments in oocytes raises the interesting question of whether the pol II transcription sites in interphase nuclei might also form them, notwithstanding their much lower transcript density? Although the small size and compact nature of these transcription sites means that their structural details have yet to be observed directly, the possibility that miniature nuclear bodies form at each active gene has recently been considered (Herzel et al. 2017). Moreover, theoretical considerations of transcriptional regulation have led to the suggestion that super enhancers reflect the formation of compartments involving transcriptional regulators, nascent transcripts, and other chromatin components (Hnisz et al. 2017).

The images of lampbrush transcription units in intact nuclei described here emphasize that in their native state, nascent RNP compartments can range in appearance from classic “thin-to-thick” gradients to complex contorted shapes. However, even though surrounded by nucleoplasm, they do not form the spherical objects characteristic of nuclear compartments previously associated with transcriptional activity, namely, histone locus bodies, Cajal bodies, and nucleoli (Handwerger et al. 2005; Zhu and Brangwynne 2015). These compartments have recently been interpreted in biophysical terms as RNP droplets that arise by liquid-liquid phase separation and are driven to a highly spherical shape by surface tension (Brangwynne et al. 2011; Zhu and Brangwynne 2015). In the case of nucleoli, in particular, it is clear that they are formed around transcribed DNA and nascent transcript RNP just as are lampbrush transcription loops. The characteristics of these extended loops as phase-separated but non-spherical nuclear structures presumably result from some type of constraint on the surface tension forces affecting compartment shape, perhaps arising from the overall lengths of pol II transcription units or properties of particular nascent RNP constituents.

In summary, the ability to visualize individual functioning transcription loops developed here has underlined how nascent RNP can be a key determinant of chromatin structure and dynamics rather than playing a passive role as simply the product of transcription.

## Electronic supplementary material

ESM 1 (PDF 2720 kb)
