# Supplementary material for: Imaging the dynamics of transcription loops in living chromosomes
Source: Chromosoma. 2018 Apr 3;127(3):361–74. doi: 10.1007/s00412-018-0667-8 (PMC6096578; doi:10.1007/s00412-018-0667-8)
Supplement: Supplementary file 1 — (PDF 2720 kb) [file 412_2018_667_MOESM1_ESM.pdf]

ONLINE RESOURCE 1

Supplementary image data: Figure 1 contains a further characterization of the distinctive CELF1-containing contorted loop locus and Figure 2 shows the dynamics of coilin in histone locus bodies of oil-isolated nuclei.

Supplementary Fig 1

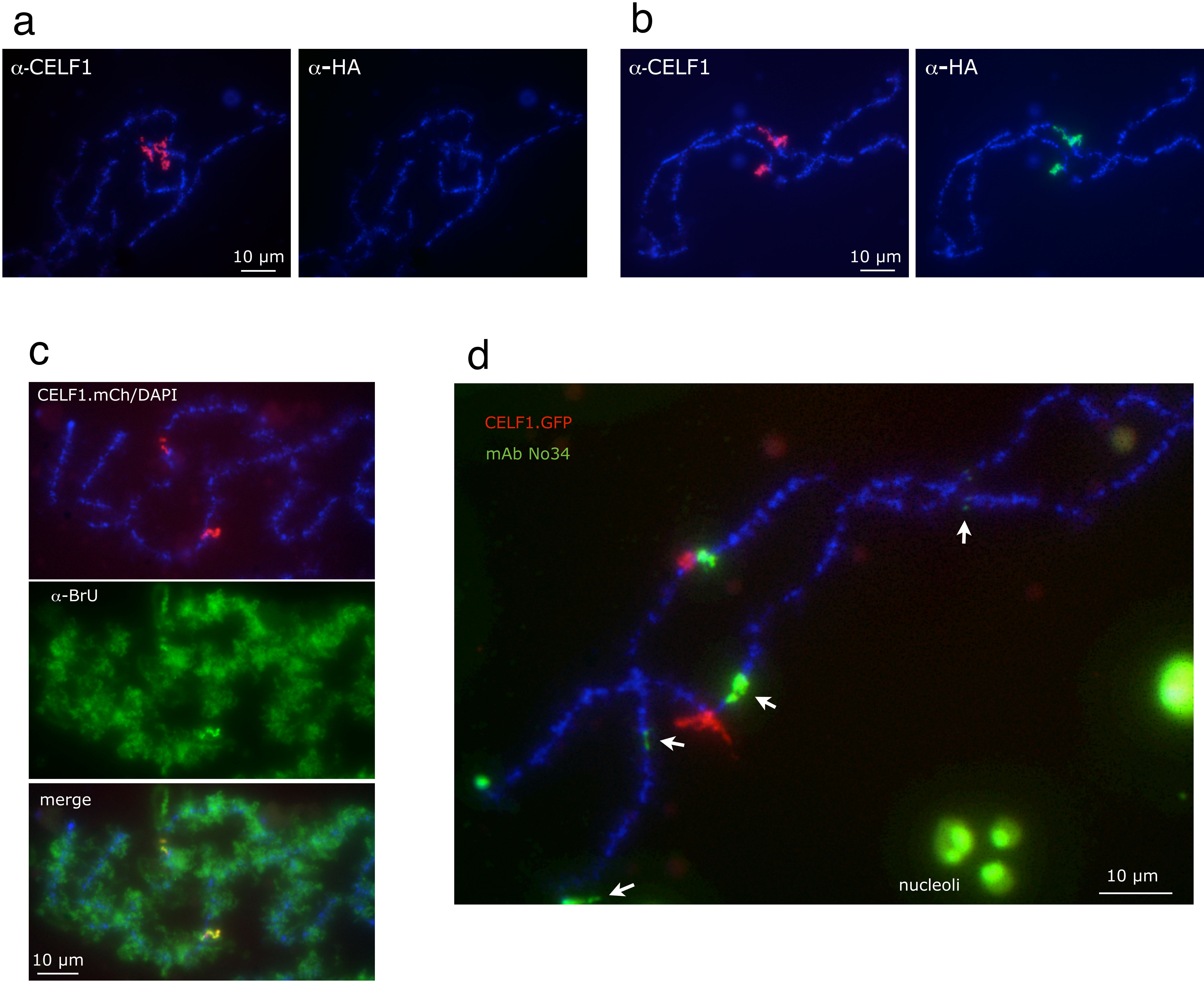

Supplementary Figure 1. Characterization of the distinctive CELF1-containing contorted loop locus.

(a) Fixed spread preparation from an uninjected oocyte immunostained with an antibody against CELF1. Fluorescent immunostaining of the contorted loop loci demonstrates the presence of endogenous CELF1 (pseudocoloured red). Co-staining with an antibody against the HA epitope is negative (pseudocoloured green). (b) Fixed spread prepared and immunostained as in (a) but from an oocyte expressing CELF1.GFP.HA (which provides additional lower level GFP fluorescence in the α-HA immunostaining channel) and The contorted loop loci are now labelled by both antibodies, demonstrating the co-targeting to these loops of endogenous and exogenous CELF1. (c) Transcriptional activity of contorted loop loci. Fixed spread prepared from an oocyte injected with Br-UTP and mCherry-tagged CELF1 mRNA and incubated overnight. Contorted loop loci are identified by mCherry fluorescence (red; top panel) and transcriptional activity is detected by immunostaining with an α-BrU antibody (green; middle panel). Merge of the two images at the bottom demonstrates Br-UTP incorporation in contorted loops (yellow). (d) The contorted loop locus targeted by CELF1 maps near the middle of the long arm of *X. laevis* LBC 7, which can be identified by its relative length and the distribution of pol III marker sites (Gall 2014; Murphy et al. 2002). DAPI-stained fixed nuclear spread from an oocyte expressing CELF1.GFP (pseudocoloured red), which identifies the contorted loops. The four pol III loci of LBC 7 (arrows) are identified by immunostaining with mAb No34, which cross-reacts with pol I and pol III (pseudocoloured green).

Supplementary Fig 2

a

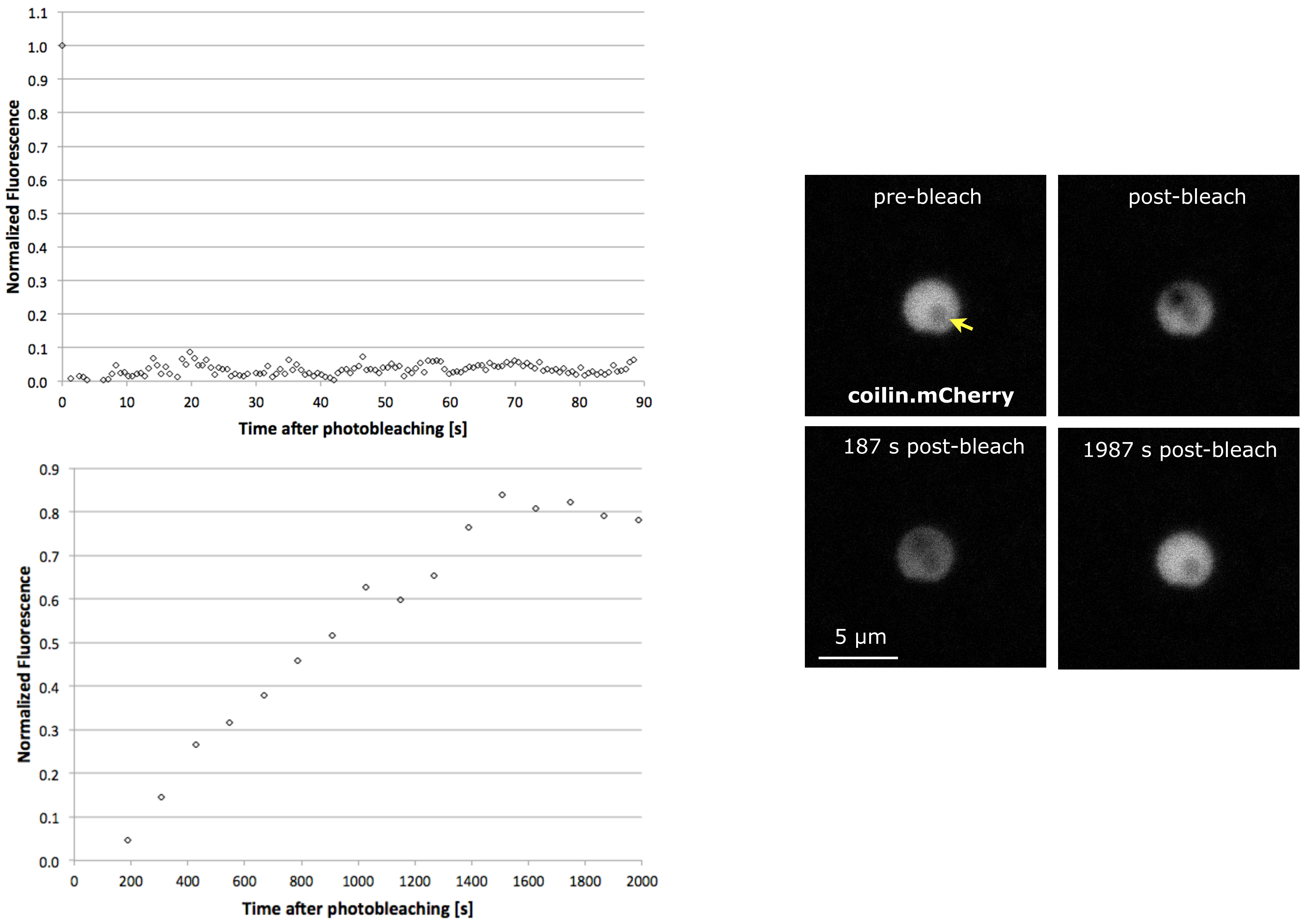

b

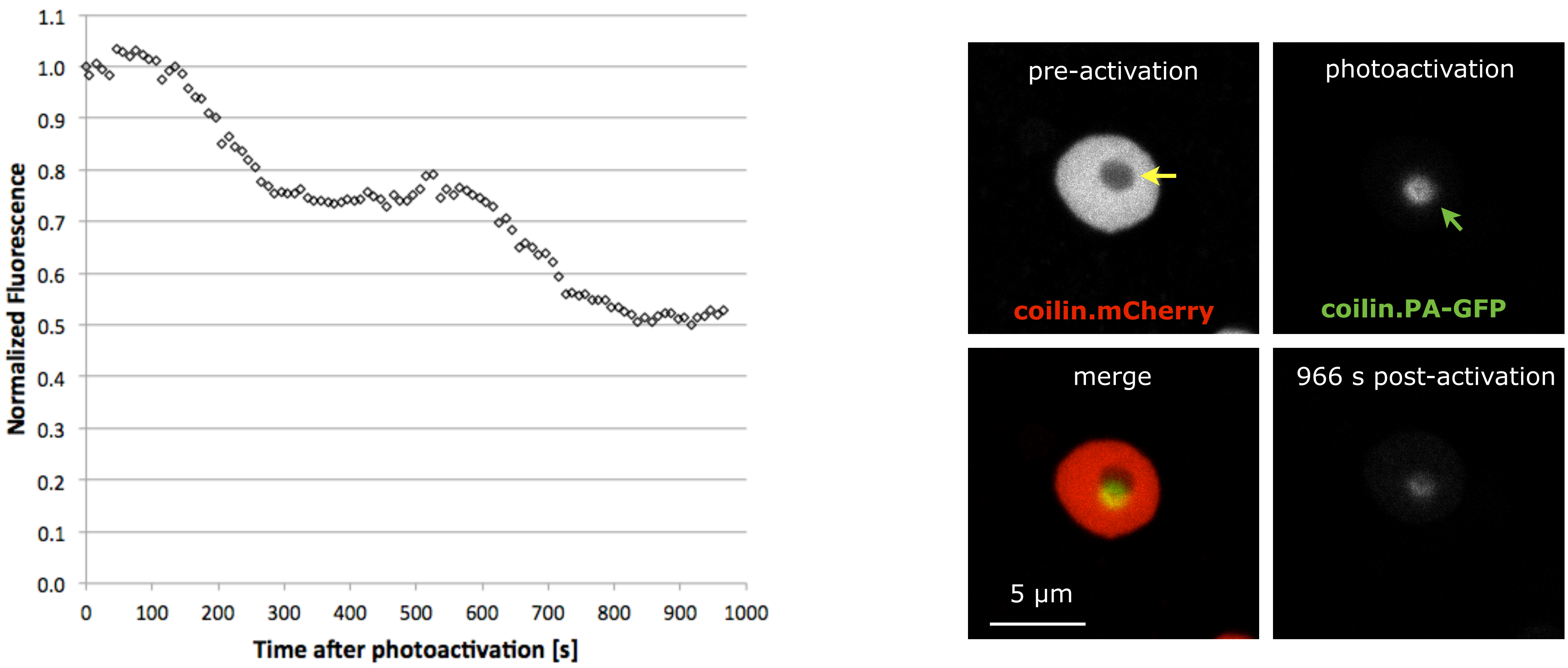

Supplementary Figure 2. Dynamics of coilin in histone locus bodies (HLBs) of oil-isolated nuclei.

These experiments are reconstructions of work previously carried out by the laboratory of Joseph Gall (Handwerger et al. 2003; Deryusheva and Gall 2004) and provide a point of comparison with the dynamics of CELF1 in contorted loops described in the main text.

(a) FRAP of coilin.mCherry in an HLB. A diffraction-limited spot was bleached in the HLB close to an internal B snurposome (arrow) and the fluorescence intensity of the bleached spot measured over time in order to follow the recovery of coilin.mCherry fluorescence. Normalized intensities were plotted against time using post-bleach images collected initially at intervals of 0.6 seconds (upper graph) and then at longer intervals of 2 minutes (lower graph). (b) Photoactivation of coilin.PA-GFP in an HLB co-targeted by coilin.mCherry. A 405 nm laser was aimed at an HLB containing an internal B snurposome (yellow arrow) *via* the pre-activation coilin.mCherry image and then used to photactivate coilin.PA-GFP in a diffraction-limited spot (green arrow). Normalized intensities of PA-GFP fluorescence were plotted over time to estimate the rate of loss of photoactivated coilin from the HLB.
